# Chimeric Antigen Receptor-T Cell Therapy for Lymphoma: New Settings and Future Directions

**DOI:** 10.3390/cancers16010046

**Published:** 2023-12-21

**Authors:** Corrado Benevolo Savelli, Michele Clerico, Barbara Botto, Carolina Secreto, Federica Cavallo, Chiara Dellacasa, Alessandro Busca, Benedetto Bruno, Roberto Freilone, Marco Cerrano, Mattia Novo

**Affiliations:** 1 Hematology Division, A.O.U. Città della Salute e della Scienza di Torino, C.so Bramante 88, 10126 Turin, Italy; bbotto@cittadellasalute.to.it (B.B.); rofreilone@cittadellasalute.to.it (R.F.); mclerico@cittadellasalute.to.it (M.C.); 2 Division of Hematology, Department of Molecular Biotechnology and Health Sciences, University of Torino, A.O.U. Città della Salute e della Scienza di Torino, C.so Bramante 88, 10126 Turin, Italy; cerranomarco@gmail.com (M.C.); facavallo4@cittadellasalute.to.it (F.C.); benedetto.bruno@unito.it (B.B.); 3 Stem Cell Transplant Center, AOU Città della Salute e della Scienza di Torino, C.so Bramente 88, 10126 Turin, Italy; csecreto@cittadellasalute.to.it (C.S.); cdellacasa@cittadellasalute.to.it (C.D.); abusca@cittadellasalute.to.it (A.B.)

**Keywords:** CAR-T cells, B-cell lymphoma, central nervous system, T-cell lymphoma, Hodgkin lymphoma, dual targeting, allogeneic CAR-T cells

## Abstract

**Simple Summary:**

Despite the impressive efficacy of chimeric antigen receptor-T (CAR-T) cell therapy in the treatment of some subtypes of lymphomas, many patients are either not eligible or relapse after treatment. Thus, great efforts are being made to further improve the depth and durability of clinical responses, as well as to expand the settings in which CAR-T cells can be employed. The present review aims to summarize those efforts, offering an overview of recent advances and future perspectives in the field.

**Abstract:**

In the last decade, anti-CD19 CAR-T cell therapy has led to a treatment paradigm shift for B-cell non-Hodgkin lymphomas, first with the approval for relapsed/refractory (R/R) large B-cell lymphomas and subsequently for R/R mantle cell and follicular lymphoma. Many efforts are continuously being made to extend the therapeutic setting in the lymphoma field. Several reports are supporting the safety and efficacy of CAR-T cells in patients with central nervous system disease involvement. Anti-CD30 CAR-T cells for the treatment of Hodgkin lymphoma are in development and early studies looking for the optimal target for T-cell malignancies are ongoing. Anti-CD19/CD20 and CD19/CD22 dual targeting CAR-T cells are under investigation in order to increase anti-lymphoma activity and overcome tumor immune escape. Allogeneic CAR product engineering is on the way, representing a rapidly accessible ‘off-the-shelf’ and potentially more fit product. In the present manuscript, we will focus on recent advances in CAR-T cell therapy for lymphomas, including new settings and future perspectives in the field, reviewing data reported in literature in the last decade up to October 2023.

## 1. Introduction

The therapeutic landscape for lymphomas has been drastically implemented in recent years. Among novel treatment options, chimeric antigen receptor (CAR)-T cells represent the most striking advance for relapsed and refractory (R/R) B-cell non-Hodgkin lymphomas (B-NHLs), along with B-cell acute lymphoblastic leukemia (ALL) and multiple myeloma [1,2,3,4,5]. Autologous lymphocytes are collected and engineered with the transduction of a CAR by a replicant-incompetent viral vector, causing T-cell recognition and killing of tumor cells by a specific antigen. CD19-targeting CAR-T cells have been introduced in clinical practice for the treatment of large B-cell lymphomas (LBCLs) since 2017. Three pivotal trials demonstrated high efficacy of three different anti-CD19 CAR-T products, namely, axicabtagene ciloleucel (axi-cel), tisagenlecleucel (tisa-cel) and lisocabtagene maraleucel (liso-cel), against R/R LBCL after two or more prior lines of therapy [6,7,8]. The three compounds, despite some structural differences that are mainly derived from a distinct costimulatory domain (CD28 for axi-cel, 4-1BB for tisa-cel and liso-cel), demonstrated comparable efficacy as a third line, achieving durable remissions in around 35–40% of patients. Thus, they obtained initial approval from international regulatory agencies for the treatment of R/R LBCL after at least two lines of therapy, with some differences in the disease histological subtypes included in the label (extended to primary mediastinal B-cell lymphomas for axi-cel and liso-cel, and to follicular grade 3B lymphomas for liso-cel) [6,7,8,9,10,11,12,13,14,15]. Many steps forward have been accomplished thereafter to ameliorate the safety and efficacy of CAR-T cell therapy and to extend its accessibility to other settings. Brexucabtagene ciloleucel (brexu-cel) is an anti-CD19 CAR-T cell product with the same structure as axi-cel but a manufacturing process that includes the removal of circulating CD19+ malignant cells. This compound demonstrated relevant efficacy against R/R mantle cell lymphoma (MCL) and its use was thus approved in this setting first in the US in July 2020 and subsequently in Europe [16,17,18]. More recently, the use of CAR-T cells has been anticipated for the first relapse/progression after frontline treatment to improve the cure rate of R/R LBCL. Three randomized phase III trials compared axi-cel (ZUMA-7 trial), liso-cel (TRANSFORM trial) and tisa-cel (BELINDA trial) with the standard-of-care (SOC) approach consisting of platinum-based salvage chemotherapy regimens plus autologous stem-cell transplantation (ASCT). The first two studies demonstrated a significant advantage for CAR-T cells (axi-cel and liso-cel) both in terms of response rates and relapse-free survival and lead to the approval of axi-cel and liso-cel as second-line treatments for refractory and early relapsed LBCL [10,11,12,13,19,20,21]. In addition, the long-term follow-up analysis of ZUMA-7 that was recently published showed an improved OS for axi-cel compared to SOC [22].

Despite the high efficacy demonstrated for B-NHLs, the rate of treatment failure remains high, with 40 to 60% of the patients being resistant or eventually relapsing after CAR-T cell treatment. Moreover, the accessibility to the treatment is restricted to selected subgroups of patients, with limitations regarding the histological disease subtype, age (in some countries), comorbidities and disease dissemination [e.g., excluding cases with central nervous system (CNS) involvement] [23]. Some of the limitations of CAR-T cell therapy regard the products themselves. First, all commercially available products for lymphoma are directed against the antigen CD19, making them ineffective in cases of CD19 expression loss [24]. Moreover, the immunosuppressive microenvironment surrounding lymphoma cells can also limit CAR-T cell efficacy. For instance, interferon signaling boosts the expression of programmed death ligand 1 (PDL-1) on cancer cells, which promotes T-cell exhaustion [25]. The quality of T-cell leukapheresis for the manufacturing of autologous CAR-T cells is also dependent on patients’ lymphocyte counts; patients’ age, disease status and recent exposure to lymphodepleting drugs can all cause insufficient cell collection and manufacturing failures [26,27].

In this review, we discuss the recent advances in the field of CAR-T cell therapy for lymphoma, focusing on new constructs under development, novel strategies to overcome treatment failure and emerging settings and disease subtypes in which they are being tested.

## 2. Upcoming Settings

### 2.1. Follicular Lymphoma

Treating follicular lymphoma (FL) patients with anti-CD19 CAR-T cells in clinical practice is already possible, since axi-cel and tisa-cel recently received Food and Drug Administration (FDA) and European Medicine Agency (EMA) approval. In the US, both products are indicated after at least 2 prior lines of therapy, while in Europe, axi-cel requires 3 previous lines [10,11,14,15].

Axi-cel was authorized based on the data from the pivotal ZUMA-5 trial. Eligible patients had R/R FL after ≥2 lines of therapy, including an anti-CD20 monoclonal antibody plus an alkylating agent. Among 124 treated patients, 36-month PFS and OS rates were 54.4% and 75%, respectively. Grade ≥ 3 cytokine release syndrome (CRS) occurred in 6% of the patients, and grade ≥ 3 immune effector cell-associated neurotoxicity syndrome (ICANS) occurred in 15% [28,29]. A propensity score matching comparison between patients with R/R FL treated in the ZUMA-5 setting and patients treated with SOC therapies strongly favored axi-cel both in terms of PFS (HR 0.28, 95% CI: 0.17–0.45) and OS (HR 0.52, 95% CI: 0.28–0.95) [30].

Tisa-cel was approved based on the ELARA trial, which also included patients with R/R FL after ≥2 lines of therapy. Ninety-four patients were treated, and the estimated 24-month PFS and OS were 57.4% and 87.7%, respectively. Toxicities were seemingly lower than those of axi-cel, with no CRS exceeding grade 2 and grade ≥ 3 ICANS of 3% [31]. Again, a propensity score matching study confirmed the stronger efficacy of CAR-T cell therapy compared to usual care both in terms of PFS and OS [32].

Preliminary results from the TRANSCEND FL trial, which extended the use of liso-cel to FL, have been recently presented. Twelve-month PFS was 80.7% and side effects were comparable to those of tisa-cel, with 1% of grade ≥ 3 CRS and 2% of grade ≥ 3 neurological events [33].

Patients experiencing progression of the disease within 24 months from first-line therapy (POD24) are still at increased risk of treatment failure after CAR-T cells and represent one of the major unmet clinical needs in the FL setting [29,31]. Indeed, the currently recruiting ZUMA-22 trial aims to compare axi-cel with SOC in the setting of high-risk R/R FL, i.e., patients failing at least one line of therapy if they experienced POD24 or otherwise after at least two lines [34].

### 2.2. Other Indolent Non-Hodgkin Lymphomas

In addition to FL cases, 28 patients with R/R marginal zone lymphoma (MZL) were treated with axi-cel in the ZUMA-5 trial. At a median follow up of 31.8 months, the median PFS was not reached. Grade ≥ 3 CRS occurred in 9% of the patients, while, surprisingly, the grade ≥ 3 ICANS rate was 36%, which was significantly higher than that observed in FL. The reason for this discrepancy is not known, but it could be related to a stronger and more prolonged CAR-T cell expansion in patients with MZL [28,29,35].

Very small reports of CAR-T cell use for Waldenstrom macroglobulinemia (WM)/lymphoplasmacytic lymphoma are available. Three heavily pretreated patients infused with two different anti-CD19 CAR-T cell products attained a clinical response; however, all three had recurrent disease between 3 and 26 months after the initial treatment [36]. More recently, a promising report of an anti-CD20 CAR-T product infused in four heavily pretreated and Bruton-tyrosine kinase inhibitor (BTKi) refractory patients has been published: the authors reported one death in complete remission 7 months after treatment due to COVID-19 infection and three patients were free from progression at 1.5–19 months, with manageable CAR-T cell-related toxicities [37].

Currently, the phase II ZUMA-25 trial is evaluating the efficacy of brexu-cel against rare R/R hematological malignancies, including WM and hairy cell leukemia [38].

Numerous basket trials including indolent lymphomas aimed at exploring novel CAR-T cell products are underway. Data resulting from the main clinical trials are summarized in Table 1 and the ongoing studies are listed in Table 2.

## 3. Extending the Setting for Large B-Cell Lymphoma

### 3.1. CAR-T Cells for Lymphomas with Central Nervous System Involvement

LBCL with CNS involvement, both isolated (primary CNS lymphoma, PCNSL) or concomitant with systemic disease (secondary CNS lymphoma, SCNSL), represents a rare subset with particularly unfavorable outcomes, especially in cases of disease relapse or frontline refractoriness [70,71]. The need for agents capable of penetrating the blood–brain barrier (BBB) has led to specific management paradigms for PCNSL and SCNSL, which therefore have been excluded from many LBCL clinical trials with novel agents [72]. The adoption of CAR-T cells in this setting was limited by the concern of potential severe neurological toxicities. This fear was supported by the identification of a brain mural pericyte cell population with CD19 antigen expression, representing a potential off-tumor target for anti-CD19 CAR-T cells [73]. Furthermore, it was unclear if CAR-T cells were capable of peripheral expansion and subsequent CNS trafficking in the absence of antigen stimulation by systemic lymphoma in cases of isolated CNS disease. Thus, PCNSLs were excluded from the three pivotal CAR-T cell trials and only a small number of patients with SCNSLs were enrolled in the TRANSCEND trial.

The capability of peripheral expansion of CAR-T cells, CNS trafficking and subsequent therapeutic activity were first highlighted in cases of ALL and primary brain solid tumors [74,75]. In 2017, Abramson et al. [76] described for the first time a case of LBCL with synchronous systemic and parenchymal brain disease recurrence treated with anti-CD19 CAR-T cells (liso-cel), obtaining a complete response with no CRS or neurotoxicity observed. Afterwards, a rising number of case series confirmed the efficacy and safety of both axi-cel and tisa-cel for R/R LBCL with CNS recurrence, comparable to non-CNS LBCL [77,78,79,80,81]. In the case series reported by Frigault M et al. [78], six of eight patients presented isolated CNS disease at the time of tisa-cel infusion and three of six obtained a clinical response, confirming the potential capability of CAR-T cells to expand even in the absence of systemic disease. Supported by the early evidence of efficacy against SCNSL, CAR-T cells have been tested against R/R PCNSL. In a retrospective analysis of five patients with R/R PCNSL treated with an academic anti-CD19 CAR-T product at City of Hope, three of five patients achieved CR at day 28 post-infusion. Of interest, in this series, CAR-T cell expansion in peripheral blood was confirmed by flow cytometry and PCR in four of five patients and CAR-T cells were detected in cerebrospinal fluid collected from one patient using flow cytometry [82]. The French Oculo-cerebral Lymphoma network confirmed the safety and efficacy of CAR-T cells in the R/R PCNSL setting in a retrospective series including nine patients treated with either tisa-cel (seven patients) or axi-cel (two patients): ICANS of any grade was observed in five patients, two with grade 3–4; at 3 months after the CAR-T cell infusion, a response was observed in six patients, five of whom obtained CRCR; and the 6-month PFS and duration of response (DOR) were 44% and 67%, respectively [83].

Given the rarity of the scenario, the available data for CAR-T cell therapy in patients with CNS lymphoma are so far limited to very small case series. Thus, Cook MR et al. [84] conducted a meta-analysis of patients with PCNSL or SCNSL treated with CAR-T cells both in clinical trials and in the real-life setting. One hundred twenty-eight patients treated with axi-cel, tisa-cel, liso-cel or other investigational anti-CD19 CAR-T compounds were included in the analysis (*n* = 30 with PCNSL, *n* = 98 with SCNSL) and the authors could confirm the efficacy and tolerability of CAR-T cells in patients with CNS disease. The CRS incidence was superimposable for PCNSL and SCNSL and was observed in around 70% of the patients, with roughly 10% being grade 3–4. ICANS occurred in 50% of the patients and a higher proportion of grade 3–4 cases was reported in SCNSL patients (26%) vs. PCNSL patients (18%), possibly because the presence of a systemic disease might translate into higher systemic inflammation and circulating cytokine levels. Fifty-six percent of PCNSL patients and 47% of SCNSL patients achieved CR as the best response and approximately 37% of the patients in both groups were in ongoing CR 6 months after the infusion. Of note, the authors observed higher toxicities and possibly superior efficacy for CD28 costimulatory domain-based products compared to 4-1BB compounds, similar to LBCL without CNS involvement.

Lastly, the German Lymphoma Group just reported the largest retrospective series of SCNSL treated with anti-CD19 CAR-T cells so far, including 28 consecutive patients, half receiving axi-cel and half receiving tisa-cel. Sixty-four percent of the patients responded, with a CR rate of 32% and a 12-month PFS of 40%, which was significantly better for patients treated with axi-cel than tisa-cel (12-month PFS of 62% vs. 19%, respectively). Of note, comparing the 28 SCNSL patients with 168 consecutive patients without CNS involvement treated with CAR-T cells, no differences in terms of survival or toxicity were observed [85].

Following the encouraging activity and safety data of CAR-T cells in patients with CNS disease, prospective trials are under development. Frigault et al. [46] conducted a phase I/II trial investigating tisa-cel in heavily pretreated patients with PCNSL who were mostly refractory to methotrexate-based regimens, BKTi and lenalidomide. No excess toxicity was observed: CRS was limited to grade 1, and, while 41.6% of the patients experienced ICANS, only one instance of grade 3 occurred. Among twelve patients infused, six obtained a CR (50%) and, with a median follow up of 12 months, three patients were still in remission at the data cut-off. The ongoing phase I CAROUSEL trial investigates AUTO1—a novel CAR-T cell product characterized by a fast off-rate CD19-binding domain—in patients with R/R PCNSL [47]. The trial includes a conditioning regimen enriched by a dose of the anti-PD1 antibody pembrolizumab, with the aim of enhancing CAR-T cell expansion, and an additional intraventricular administration of AUTO1 thorough the Ommaya reservoir for patients not achieving a response after the intravenous infusion. A preliminary analysis showed a response in four out of five patients infused, with two patients in continuous CR at 6 months; one patient experiencing disease progression at 1 month post-infusion demonstrated a transitory response after the intraventricular treatment rechallenge. Li T et al. [48] conducted a study with a “cocktail” strategy of anti-CD19 and anti-CD22 CAR-T cell infusion in a small number of patients with R/R B-NHL with CNS involvement, obtaining short-lasting responses in two out of five patients. Thereafter, the same group tested a sequential approach of CD19/CD22 CAR-T cell cocktail infusion following ASCT in 13 patients with CNS lymphoma, achieving rather high rates of responses (ORR 82%, CR 55%), which appeared to be durable (median DOR 14.4 months) [86].

Data on CAR-T cells for MCL with CNS involvement are limited to the case of a refractory patient who achieved CR persisting 3 months after brexu-cel infusion and who experienced only reversible toxicities [87].

Globally, most available data about CAR-T cells in lymphomas with CNS disease showed an encouraging efficacy and a manageable toxicity profile, strongly supporting their use in this setting. Hopefully, future dedicated prospective trials will confirm these results, leading to the extended authorization of CAR-T cells for these subsets.

### 3.2. Frontline Adoption of CAR-T Cells

The anticipation of CAR-T cell therapy from the third to second line demonstrated a significant benefit for refractory or early relapsed patients with LBCL in two of the three randomized phase III trials mentioned above, leading to the approval of axi-cel and liso-cel in this setting [19,20,21]. Following these favorable results, CAR-T cells have been explored as first-line therapy for high-risk LBCL patients. Axi-cel has been first tested as part of the frontline treatment in the phase II single arm ZUMA-12 trial [88]. In the study, 40 patients with high-risk LBCL, defined by an IPI score ≥ 3 or double/triple-hit histology and with a PET-guided incomplete response after two courses of R-CHOP (based on the Deauville criteria) [89], were treated with axi-cel. The trial met the primary end-point, reaching high rates of response (ORR 89%), with a 78% CR rate and 73% of the patients in a continuous response after a median follow-up of almost 16 months. Next, the phase III ZUMA-23 trial has been designed to prove the real benefit of axi-cel over the SOC in the frontline setting [90]. Treatment-naïve patients with very high-risk LBCL, defined by an IPI score ≥ 4, are randomized to receive axi-cel or continue with SOC following a first cycle of chemoimmunotherapy (R-CHOP or rituximab, etoposide, prednisolone, vincristine, cyclophosphamide, and doxorubicin [R-EPOCH]). The trial is currently ongoing and will hopefully demonstrate whether a frontline CAR-T cell approach may be the best option for selected high-risk LBCL patients.

## 4. New Targets for B-NHLs: Multispecific CAR-T Cells

Long-term PFS of patients with aggressive NHL treated with anti-CD19 CAR-T cells remains around 30–40% [91,92] and about a third of the patients who relapse show CD19 negativity in tumor biopsy samples due to selection of CD19-negative clones and antigen downregulation [24]. Thus, multi-targeted CAR-T cell constructs are being developed to overcome this issue [93,94].

Multiple approaches have been adopted to target more than one tumor antigen. Different single targeting CAR-T cell products can be infused in the same patient at the same time or sequentially. T-cells can also be transduced by different vectors at the same time, creating a population of cells that express different combinations of CARs. A single bicistronic vector encoding two different CARs can be used to create a single T-cell population expressing both CARs. Lastly, a single vector can also be used to encode two CARs on the same receptor (tandem CAR).

**Figure 1:**
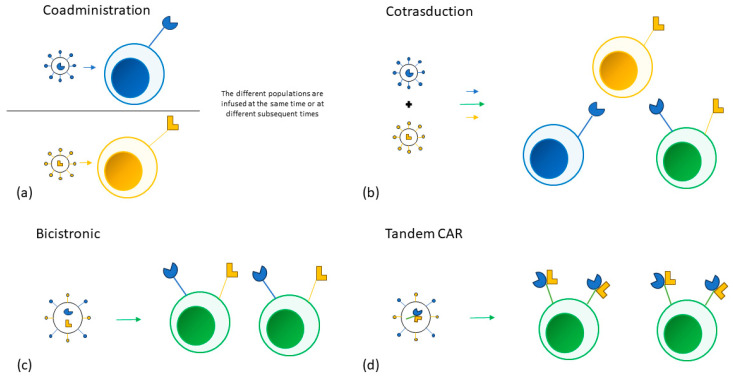
(**a**) Two different single targeting CAR-T products are separately manufactured and are then infused in the same patient at the same time or at different subsequent times. (**b**) T-cells are transduced by two different vectors at the same time, creating three distinct T cell populations. (**c**) A single bicistronic vector encoding two different CARs is used to transduce a single T-cell population. (**d**) A single vector encoding two different CARs on the same receptor is used to transduce a single T-cell population.

No data comparing the different approaches is available [95]. The different strategies could also be combined in the treatment of the same patient and are represented in Figure 1.

The most studied approach involves bispecific CAR-T cell constructs: anti-CD19/CD22 and anti-CD19/CD20 CARs have been studied in both preclinical and clinical settings.

CD20 has been a target of immunotherapies since the advent of rituximab. It is expressed almost exclusively in B-cells, and its expression was found to remain stable even in the context of CD19-negative relapses [24,96]. Two phase I/II trials reported encouraging results from a total of 103 patients with R/R B-NHL. In the study by Zhang et al. [39], a CR rate of 70% was obtained and, after a median follow-up of 27.7 months, the median PFS was 27.6 months and median OS was not reached. Nine patients (10%) experienced grade ≥ 3 CRS and two (2%) had grade ≥ 3 ICANS. Three patients died of treatment-related complications. Seven out of nine patients who were previously treated with anti-CD19 CAR-T cells responded to the treatment. Of note, 12 of the patients who relapsed were biopsied: only one demonstrated a double negative CD19-/CD20- disease and no loss of either CD19 or CD20 alone was observed. Shah et al. [40] reported results from the phase I dose escalation and expansion trial of the bispecific CD19/CD20 LV20.19 CAR-T product. Among the 16 patients who received the maximum dose (2 × 10^6^ cells/kg), the ORR and CR rate were 100% and 92%, respectively. After a median follow-up of 31 months, the median PFS was 15.6 months and median OS was not reached [41]. The construct was well tolerated, with grade ≥ 3 CRS occurring in one patient (5%), grade ≥ 3 ICANS in three patients (14%) and no treatment-related death was observed. No relapses due to antigen escape were reported.

Similarly to CD20, the CD22 antigen is expressed almost exclusively in B-cells and plays a relevant role in B-cell survival [97]. Wang et al. [42] reported the results of a sequential infusion of anti-CD19 and anti-CD22 CAR-T cells in 36 patients with R/R B-NHL. The ORR was 72%, the CR rate was 50%, and grade ≥ 3 CRS occurred in eight patients (21%). Two patients died due to treatment-related complications. With a median follow-up of 14.4 months, median PFS and OS were 9.9 months and 18.0 months, respectively. Seven out of eighteen relapsed patients were biopsied and no antigen-negative relapse was identified.

Further studies showed the relevant activity of bispecific anti-CD19/CD22 CAR-T constructs. Zhang et al. [43] treated 32 patients with R/R B-NHLs, obtaining an ORR of 79.3%, and a CR rate of 34.5%. PFS and OS rates at 12 months were 40.0% and 63.3%, respectively. Grade ≥ 3 CRS occurred in nine patients (28.1%) and grade ≥ 3 ICANS in four (12.5%). One patient died because of CRS. Similar results were obtained in a population restricted to R/R LBCL patients. Wei et al. [44] reported encouraging response rates in a cohort of 16 patients (87.5% ORR, 62.5% CR). Two-year PFS and OS were 40.2% and 77.3%, respectively. Toxicities were manageable: only one patient experienced grade ≥ 3 CRS and no ICANS was observed. Spiegel et al. [45] reported the results of a phase I study including 22 patients: the ORR was 62% with a CR rate of 29%. Grade ≥3 CRS occurred in one patient (5%), as well as ≥ grade 3 neurotoxicity. The median PFS was 3 months and median OS 22.5 months. Three out of fourteen patients who were biopsied at progression presented low levels of CD19; the expression of CD22 was maintained in the six patients who had a high pretreatment expression of CD22.

Other targets have been studied in preclinical in vitro and in vivo settings, namely, CD19/CD79b (CD79b is already being employed as a target by available antibody–drug conjugates), CD19/CD38, CD19/CD123 and CD19/CD37 CARs, all showing promising antitumoral activity [98,99,100,101].

Increasing the number of target antigens beyond bispecific CAR-T cells should further reduce the risk of antigen escape and thus of disease relapse. In vitro and in vivo experiments demonstrated increased CAR-T cell expansion and improved antitumor effects through the sequential administration of single targeting anti-CD19, anti-CD20 and anti-CD22 cells. Compassionate treatment of two patients with Burkitt lymphoma and LBCL seems to confirm the observation, showing delayed re-expansion of previously infused cells upon the administration of subsequent products. CAR-T cell-specific toxicities were manageable, consisting of mild to moderate CRS, with no ICANS observed [102].

No clinical data are available regarding efficacy and safety of trispecific constructs in clinical settings, while preclinical data in mice showed strong activity even against cells negative for one of the three targeted antigens [103].

Although available data regarding multispecific CAR-T cells show promise in reducing CD19-negative lymphoma relapse, they are still limited to small phase I trials and some early phase II trials. Moreover, treatment with multispecific CAR-T cell constructs does not have an impact on reducing relapse due to reasons other than antigen escape. To address the latter limitation and avoid T-cell exhaustion, the association of bispecific CAR-T cells with anti PD-1 monoclonal antibodies has been explored, with encouraging early results [104]. Even if not commercially available nowadays, the costs of production of multispecific CAR-T cells may represent a limit to their employment in clinical practice.

## 5. New Lymphoma Settings

### 5.1. Hodgkin Lymphoma

Despite the success of frontline therapy, 20–30% of patients with classical Hodgkin lymphoma (cHL) progress or relapse during the course of their disease history. Salvage with high-dose chemotherapy followed by ASCT remains the standard of care in second-line treatment. Targeted therapy with the anti-CD30 antibody-drug conjugate brentuximab vedotin and immunotherapy with anti PD1/PDL1 are currently considered the best options for subsequent relapses and, more recently, their introduction in combination with chemotherapy regimens in the upfront setting further improved outcomes for patients with advanced stage disease [105,106,107]. Nevertheless, a significant fraction of patients needs alternative approaches.

cHL is characterized by a reduced number of malignant Hodgkin and Reed–Sternberg (HRS) cells and an abundance of inflammatory and immune cells, including reactive T- and B-lymphocytes, macrophages, granulocytes and fibroblast-like cells. CD30, a member of the TNF superfamily, is selectively over-expressed in HRS cells with very low expression in normal tissues, and therefore it is considered a promising target for novel treatments.

The first phase I trial experimented with an anti-CD30 CAR-T cell product in 18 heavily pretreated patients [49]. The ORR was 39% (all partial responders), with 28% of patients showing stable disease at two months and a median PFS of 6 months. All patients experienced grade 1 or 2 CRS at the time of the infusion that resolved without specific treatments. Delayed toxicities mostly occurred 2–4 weeks after cell infusion without any evidence of ICANS; no treatment-related deaths occurred during the study.

In another phase I/II trial of an anti-CD30 CAR-T cell product in R/R cHL, 41 patients underwent lymphodepletion with different regimens of bendamustine alone, bendamustine–fludarabine or cyclophosphamide–fludarabine [52]. All patients were heavily pretreated, with a median of seven prior lines of therapy, including brentuximab vedotin, anti-PD1 antibodies, and ASCT or allogeneic stem cell transplantation (allo-SCT). CRS was observed in 10 patients, all events being grade 1, and no case of ICANS was reported. Among the 32 patients who received fludarabine lymphodepletion, the ORR was 72% and 19 patients (59%) achieved CR. One-year PFS and OS were 36% and 94%, respectively.

Another small clinical trial enrolled nine R/R CD30+ lymphoma patients [six with HL, three with anaplastic large cell lymphoma (ALCL)] who received a third-generation CAR-T cell therapy with both CD28 and CD137 as costimulatory domains following lymphodepletion with fludarabine and cyclophosphamide. Seven patients achieved CR, which was durable in three patients who were still in remission after 2 years, and the median PFS was 13 months. Six patients experienced CRS, grade 1–2 in four patients, and no ICANS was observed [50].

Voorhees and colleagues explored potential factors associated with a favorable PFS after anti-CD30 CAR T-cell therapy in R/R cHL patients [108]. Patients with a higher (>60 mL) metabolic tumor volume (MTV) by PET before CAR T-cell therapy had a lower 1-year PFS (14%) compared to those with a low MTV (58%). In contrast, bridging therapy, anti-CD30 CAR-T cell expansion/persistence and the percentage of CD3+ lymphocytes over the first 6 weeks of therapy did not impact PFS.

The same authors evaluated the role of checkpoint inhibitors in patients with progressive disease after CAR-T cell therapy, showing a clinical benefit in all patients, including those without a previous response to anti-PD1. Possibly, these drugs could reactivate CAR-T cells that persisted after the initial infusion, which might explain this finding [109].

Globally, CAR-T cell therapy has demonstrated good response rates and an acceptable safety profile in small clinical trials. However, larger controlled studies with longer follow-up are required to confirm durability of responses in cHL patients.

### 5.2. T-Cell Lymphomas

Despite their rapidly expanding use against B-cell malignancies, the development of CAR-T cell therapy for T-cell neoplasms is rather challenging and no products are currently available for this subset of lymphomas.

Most antigens tested as CAR-T cell targets for T-cell neoplasms (such as CD3, CD5, and CD7) are also expressed by normal T-cells [110]. Therefore, the lack of a tumor-specific antigen can cause the eradication of normal T-lymphocytes by CAR-T cell treatment, leading to T-cell aplasia, a possibly life-threatening phenomenon [111,112].

Additionally, the expression of their own target antigens by CAR-T cells leads to mutual killing, also known as fratricide, resulting in impaired persistence and efficacy [113].

Although less common than in ALL, circulating tumor cells might be found in peripheral blood. Therefore, T-cell collection could be contaminated with malignant cells, which can be erroneously transduced, as reported in a patient who relapsed by expressing anti-CD19 CAR-tumor cells [114].

One of the fundamental challenges in developing effective CAR-T cell therapies for T-cell lymphomas lies in the identification of suitable target antigens. The ideal target should be specific for the malignant cells and not expressed on normal cells, but T-cell lymphomas originate from mature T-cells and lack a clear distinctive cell surface marker. Although most clinical trials on T-cell malignancies have focused on T-ALL, here, we summarize some of the potential target antigens being explored by researchers for T-cell lymphomas. Several clinical trials are currently ongoing and recruiting patients almost exclusively in China [115].

CD7 is probably the most studied target in T-cell malignancies. Several strategies, including CRISPR-Cas9, natural selection, and endoplasmic reticulum retention, have been developed to block CD7 expression on CAR-T cells and avoid fratricide, since CD7 does not appear essential for T-cell development or function [64,116,117]. The so-called IntraBlock donor-derived anti-CD7 CAR-T cells have demonstrated promising safety and efficacy in patients with T-ALL in a phase I clinical trial [118]. However, few conclusive experiences in T-cell lymphomas have been reported so far and several trials (mainly phase I) are still ongoing. In a recently published experience, ten patients with R/R T-cell malignancies, including one patient with angioimmunoblastic T-cell lymphoma (AITL) and one with mycosis fungoides, were treated with anti-CD7 autologous and allogeneic CAR-T cells. The patient with mycosis fungoides achieved PR on day 65 and relapsed on day 180. No details on the other patients are available, but overall, a remarkable efficacy (ORR 70% at 6 months median follow-up) and an acceptable toxicity were shown, especially for patients with allogeneic products [54].

The results of a clinical trial with CD5-directed CAR-T cells in four T-ALL patients and five T-NHL patients enrolled were reported in 2019. Four out of nine evaluable patients obtained an objective response, including three with a CR. Two of them did not proceed to planned allo-SCT and relapsed at 6 weeks and 7 months post-infusion, respectively. Another patient with AITL who received a second CAR-T cell infusion and underwent allo-SCT in tandem was still in CR 4 months thereafter [53]. Based on these results, allo-SCT after CAR-T cell bridging may represent a strategy to acquire deeper and longer remissions. Moreover, toxicity data were promising, with few CRS events and short term cytopenias.

A case of Sezary syndrome treated with CD4-directed CAR-T cells has been described [119]. The patient obtained a CR with low-grade CRS and no other adverse events observed. Although targeting CD4 appears effective, serious CD4+ T-cell aplasia can develop.

Clinical trials with anti-CD30 directed CAR-T cells included mainly patients with R/R cHL and ALCL. In a first report, one patient with ALK+ ALCL received four infusions of CAR-T cells and achieved a CR that lasted for 9 months. However, no response was observed in another patient with cutaneous ALK- ALCL enrolled in the trial [51]. In another phase I trial, the only patient diagnosed with cutaneous ALCL obtained a CR after two infusions of anti-CD30 CAR-Ts [49]. Similar results were described in a different trial, in which other two ALCL patients obtained a CR [120]. Finally, a patient with R/R enteropathy-associated T-cell lymphoma (EATL) treated with anti-CD30 CAR-T cells showed a 24-month lasting remission [121]. Of note, adverse events were limited throughout the trials, indicating a promising safety and efficacy profile of anti-CD30 CAR-T cells, with a great potential for ALCL treatment. However, a cHL patient died of severe CRS, raising some concerns about safety, with the need for a better selection of the timing and dosing of the treatment [120].

CD37 is expressed on several hematological malignancies, and testing its presence before therapy would be necessary to select suitable patients. The only ongoing trial of CD37-directed CAR-T cells (NCT04136275) included a single patient with cutaneous T-cell lymphoma (CTCL). Despite poor ex vivo proliferation, this patient achieved CR a month after infusion. Of note, all infused patients (*n* = 4) experienced CRS and ICANS, and two developed prolonged pancytopenia with marrow aplasia that required allo-SCT [55].

The expression of T-cell receptor beta-chain constant domains 1 and 2 (TRBC1 and TRBC2) is mutually exclusive [122]. This targeting strategy can therefore spare a proportion of the normal T-cell compartment. An ongoing phase I-II trial with AUTO4, a TRBC1 directed autologous CAR-T cell therapy for patients with TRBC1-positive PTCL, is showing promising results. Of the twelve patients enrolled, seven were diagnosed with PTCL-NOS, four with AITL and one with ALCL. At one month, five patients were in CR, one achieved a PR and three patients did not respond. Interestingly, three of four patients treated with the highest dose of AUTO4 achieved a CR. The safety profile was acceptable with transient cytopenias, one grade 3 CRS, no ICANS and no dose-limiting toxicities [56,123]. Another trial of anti-TRBC1 CAR-T cells in patients with R/R TRBC1+ T-cell malignancies is currently recruiting [124].

## 6. ‘Off-the-Shelf’ Products for NHLs: Allogeneic CAR-T and CAR-NK Cells

Despite their efficacy in lymphoma, several challenges still hinder patients’ access to autologous CAR-T cells. In addition to the known efficacy and safety issues, factors such as high costs, a complex manufacturing process, and a vein-to-vein time of approximately three to four weeks required for personalized T-cell production pose significant barriers [125,126,127]. As a consequence, efforts are underway to overcome these obstacles through the development of allogeneic CAR-T cells (allo-CAR-Ts), often referred to as ‘off-the-shelf’ CAR-T cells, and the exploration of alternative effector cells for CAR therapy.

### 6.1. Allogeneic CAR-T Cells

In contrast to autologous CAR-T cells, off-the-shelf products offer several potential advantages. First, T-cells are collected from healthy donors, thus avoiding the potential detrimental effects of cancer or and cytotoxic agents. Additionally, large quantities of allo-CAR-Ts can be derived from a single donor, enabling the creation of readily available batches of preserved CAR-T cells for immediate patient access. The increased availability of the product may result in a reduced need for bridging chemotherapy and lower costs [128,129]. Finally, since allo-CAR-Ts can be created from T-cell subsets that may confer properties such as memory or stemness, a better persistence might be obtained [127,130].

Despite the potential benefits of allo-CAR-Ts over autologous CAR-T cell products, if immune cells are sourced from MHC-mismatched donors, these products may lead to graft rejection and to the development of graft-versus-host disease (GVHD). Thus, new sources of T-cells for allogeneic approaches have been explored both in preclinical and clinical studies, including virus-specific T-cells, genetically modified conventional T-cells, and non-conventional T-cells [127,131].

The adoption of virus-specific T-cells represents a promising way for mitigating the GVHD risk, given their established role in treating post-transplant viral infections [132]. The safety of this approach was demonstrated in a phase I basket trial involving patients with various B-cell malignancies who received CAR virus-specific T-cells, in which severe GVHD did not occur [133].

T-cell genetic modification offers another strategy to address GVHD and rejection concerns by removing endogenous molecules such as αβ T-cell receptors (TCR) and MHC. In an early clinical study, two infants with R/R ALL were successfully treated with universal CAR-T cells, a product in which CD52 and αβ TCR were disrupted through a transcription activator-like effector nuclease (TALEN) gene editing technique [134]. While TCR suppression mitigated the GHVD risk, genetic disruption of CD52 expression allowed the adoption of the anti-CD52 alemtuzumab monoclonal antibody as part of the lymphodepletion without affecting CAR-T cell activity. More recently, a CRISPR/Cas9 base-edited anti-CD7 CAR-T product characterized by a triple CD52/CD7/βTCR gene suppression pattern showed efficacy against R/R T-ALL [135]. In this case, the gene inactivation of CD52, CD7 and the β chain of the αβ TCR favored the evasion of lymphodepleting therapy, fratricide and GVHD, respectively.

Furthermore, ongoing research is exploring the introduction of CARs into various T-cell subtypes. Among these, gamma delta T-cells (γδ T-cells), constituting approximately 5–10% of the T-cell population, stand out as a promising choice for off-the-shelf CAR-T cell production. This is attributed to the MHC-independent nature of γδ TCR, which reduces the GVHD risk [127]. Additionally, the potential of CAR-γδT cells in treating solid tumors has been showed in a proof-of-concept study, in which CAR-γδT cells exhibited increased anti-tumor activity while preserving their intrinsic γδ T-cell function [136].

### 6.2. CAR-NK Cells

Natural killer (NK) cells and macrophages are emerging as highly promising candidates for the development of next-generation off-the-shelf CARs, thanks to their advantageous characteristics. NK cells and macrophages are innate immune system components capable of directly recognizing target cells independent of MHC. Importantly, they do not trigger GVHD and their ability to recognize tumor cells even when MHC molecules are downregulated might prevent antigen escape. Moreover, NK cells can be sourced from various allogeneic origins, such as induced pluripotent stem cells or umbilical cord blood (UCB), as NK cell activation does not rely on the MHC pathway [137].

The efficacy of CAR-NK cells has been demonstrated in preclinical models across a range of hematological and solid tumors [138]. Furthermore, in a phase I/II clinical study involving 11 patients with CD19-positive hematological malignancies, promising antitumor effects without significant toxicities were reported following allogeneic UCB-derived CAR-NK cell administration [63].

Another compelling option with unique attributes is represented by macrophages. First, they can eliminate tumor cells through selective phagocytosis and can also present antigens to T-cells, thereby activating adaptive immunity. In addition, macrophages are the most abundant and highly infiltrated innate immune cells within the tumor microenvironment. Finally, they are able to produce chemokines and cytokines, making them significant immunomodulators capable of reshaping the suppressive tumor microenvironment [139,140]. However, no clinical trials involving CAR macrophages (CAR-M) targeting lymphomas are active to date and preliminary data are limited to solid tumors [141].

### 6.3. Clinical Experiences with NHLs

The first clinical trial of allo-CAR-Ts (phase I ALPHA study, NCT03939026) showing the safety and feasibility of this approach included patients with R/R LBCL or FL after at least two lines of therapy. These patients received a single infusion of healthy donor-derived CAR-T cells without prior lymphodepletion chemotherapy. The ALLO-501 CAR-T cells are genetically modified anti-CD19 CAR-T cells with disrupted TCR alpha and CD52 genes, thus reducing the risk of GVHD and allowing the use of a humanized anti-CD52 mAb (ALLO-647) for selective and transitory host lymphodepletion. Forty-six out of forty-seven enrolled patients received ALLO-501, and the treatment was initiated rapidly, with a median time of 5 days from enrollment to therapy start. No GVHD was reported and limited ICANS and CRS were observed. Cytopenias occurred in 82.6% of the patients, and grade ≥ 3 infections were observed in 23.9% of the cases, similar to what is observed with autologous CAR-T cells. The 6-month CR rate for LBCL patients was 36.4% [57]. The ongoing ALPHA2 study (NCT04416984) is evaluating ALLO-501A (the next-generation product based on ALLO-501 results and lacking the rituximab recognition domains) with either a single or an additional consolidative dose of treatment. In this latter group, patients with ≥SD at day 28 received consolidation with a second ALLO-647 and ALLO-501A cell infusion. A comparable safety profile and improved efficacy with consolidation dosing were shown, including CAR-T cell persistence and deepening of responses (ORR 58%, CR 42%) [58].

Another allo-CAR-T cell product, PBCAR0191, is being assessed in R/R B-NHL patients who have received at least two prior lines of therapy (NCT03666000). Preliminary data reported a rapid time from eligibility confirmation to PBCAR0191 infusion (median of 7 days). PBCAR0191 exhibited dose- and lymphodepletion-dependent cell expansion kinetics and a promising toxicity profile with no GVHD or grade ≥ 3 CRS events and a single case of grade 3 ICANS among the 15 patients infused. The CR rate at day ≥28 ranged from 33% for patients receiving standard lymphodepletion to 80% for those receiving enhanced lymphodepletion. A portion of responding patients underwent allo-SCT, and the assessment of the response duration is ongoing [59].

Finally, a phase I/II study with CTX110, a CRISPR/Cas9-based allo-CAR therapy with the insertion of the CAR construct into the TCR alpha gene locus and the disruption of endogenous TCR and MHC, in patients with R/R LBCL is currently underway (NCT04035434). As of April 2022, data from 34 patients demonstrated a safety profile comparable to autologous CAR-T cell therapy, with a 67% ORR and a 41% CR rate [60].

A list of currently active or recruiting clinical studies of allo-CARs, including CAR-NK cells, is shown in Table 2.

## 7. Other Strategies for CAR-T Cell Manufacturing Improvement

The vector choice for T-cell transduction can also impact CAR activity and clinical outcomes: traditional “always-on” promoters, such as those carried by retroviral vectors, more commonly lead to tonic signaling and overstimulation of CAR-T cells, which can lead to exhaustion and disease relapse. Enhancing immune cell fitness and limiting or interrupting the interaction between CAR-T cells and target antigens are promising strategies to overcome this limitation [142,143,144].

Both these objectives can be reached thanks to the use of next-generation gene modification strategies, such as the use of lentiviral vectors, which do not rely on cell division and have a higher transduction efficiency, or with nonviral methods, such as CRISPR/Cas9 technology. Disruption of the regulation of DNA methylation in CAR-T cells has resulted in enhanced T-cell proliferation and a more prolonged antitumor response, as was first observed in a clinical trial as a result of casual disruption via lentiviral integration of the gene TET2. This observation was later confirmed in a preclinical model by knocking out the gene DNMT3A [145,146]. The downregulation of the activity of specific genes or the overexpression of target transcription factors can lead to a substantial improvement in T-cell potency, expansion and persistency, and many potential targets are being explored [147]. Moreover, numerous regulatable platforms are being developed: the possibility of temporarily “switching off” CAR-T cell activity via drug-sensitive promoters or other means aims to provide rest periods that could prevent T-cell exhaustion. Furthermore, the engineering of lentiviral vectors to carry genes encoded under activation-inducible promoters, which are activated only in presence of specific tumor antigens, could help further reduce the risk of disease resistance due to an immunosuppressive microenvironment or T-cell exhaustion [144,147].

## 8. Conclusions

In the last decade, anti-CD19 CAR-T cells reshaped the treatment paradigm for patients with R/R LBCL and MCL, and many steps forward have been made since the first approval in these settings. Axi-cel and liso-cel have already moved to the second line for refractory and early relapsed patients with LBCL and, more recently, CAR-T cell access has been extended to R/R FL. Several reports demonstrated the efficacy and safety of CAR-T cells in patients with CNS lymphoma and could hopefully support the extension of the regulatory approval to this setting. The use of axi-cel as part of frontline treatment for patients with adverse risk LBCL showed promising results in the phase II single arm ZUMA-12 trial, and the ongoing phase III randomized-controlled ZUMA-23 trial will prove whether this approach will be the new SOC for selected patients with very-high-risk LBCL. New strategies are actively being tested to improve CAR-T cell efficacy and accessibility. Dual targeting CD22/CD19- and CD20/CD19-CAR-T cell products showed significant activity against R/R B-NHLs, emerging as a promising strategy to overcome tumor antigen escape mechanisms. Allogeneic CAR products represent an attractive alternative to autologous CAR-T cells thanks to their ‘off-the-shelf’ nature and potential increased antitumor activity, but further data are necessary to thoroughly assess the long-term implications. Finally, early studies demonstrated promising efficacy of CAR-T cells against new disease subtypes, including R/R cHL and, despite the challenges, T-cell lymphoma.

The world of CAR-T cell therapy is rapidly evolving. In the next few years, we will likely witness empowered CAR-T cell products with extended accessibility for novel settings.

## Figures and Tables

**Table 1:** Efficacy and safety profile of recently approved and novel CAR-T cell products.

CAR-T			Disease	Line of Therapy (*n*)	Patients (*n*)	Efficacy			Toxicity	
Product (study ref)	Target	Costimulatory domain				ORR(CR)	PFS	OS	CRS (grade ≥ 3)	ICANS (grade ≥ 3)
B-cell non-Hodgkin lymphomas										
Axi-cel (ZUMA-5) [29]	CD19	CD28	FL	>1	124	94 (79)%	3-year 54%	3-year 75%	(6)%	(15)%
Tisa-cel (ELARA) [31]	CD19	4-1BB	FL	>1	94	86 (68)%	2-year 57%	2-year 88%	49 (0)%	3 (1)%
Liso-cel (TRANSCEND) [33]	CD19	4-1BB	FL	>1 POD24/>2	130	97%/94.1% in 101 pts, line of therapy ≥ 3	1-year 81%	NA	58 (1)%	15 (2)%
Axi-cel (ZUMA-5) [28,29]	CD19	CD28	MZL	>1	28	83 (63)%	median 17 m	2-year 70%	(9)%	(36)%
TanCAR7 [39]	CD19/CD20	4-1BB	BNHL	>1	87	78 (70)%	media 28 m	median not reached	70 (10)%	17 (2)%
LV20.19 [40,41]	CD19/CD20	4-1BB	BNHL, CLL		22	82 (64)%	median 16 m	median not reached	64 (5)%	32 (14)%
CART-cocktail ° [42]	CD19, CD22 (sequential infusion)	CD28, 4-1BB	BNHL	>1	36	72 (50)%	median 10 m	median 18 m	100 (21)%	13 (0)%
CD19/22 CAR [43]	CD19/22	4-1BB	BNHL		32	79 (34)%	1-year 40%	1-year 63%	91 (28)%	16 (12)%
CD19/CD22 CAR [44]	CD19/CD22	4-1BB	DLBCL	>1	16	87 (62)%	2-year 40%	2-year 77.3%	100 (6)%	0%
D19-22.BB.z-CA [45]	CD19/CD22	4-1BB	LBCL	>2	22	62 (29)%	median 3 m	median 22 m	76 (5)%	43 (5)%
Tisa-cel [46]	CD19	4-1BB	PCNSL	>1	12	58 (50)%	1-year 25%	1-year 58%	58 (0)%	42 (8)%
AUTO1 (I ± I-VEN) (CAROUSEL) * [47]	CD19	4-1BB	SCNSL	>1	5	2/4 (2/4) •	NR	NR	4 (0) •	2 (2) •
CAR-T cocktail ° [48]	CD19, CD22	4-1BB, CD28	SCNSL, PCNSL	>1	5	5/5 (1/5)	median 3 m	6-month 100%	5/5 (0) •	2/5 (1) •
Hodgkin lymphoma, T-cell lymphomas										
CART-30 [49]	CD30	4-1BB	HL, ALCL	>2	18	39 (39)%	median 6 m	ns	100% (NR)	ns
CD30 CART [50]	CD30	4-1BB, CD28	HL, ALCL	>2	9	78 (78)%	median 13 m	nr	67 (20)%	0%
CD30.CAR-T [51]	CD30	CD28	HL, ALCL	≥3	9	3/9 •	NR	NR	0%	0 (0)%
CD30.CAR-T [52]	CD30	CD28	HL	>2	41	72 (19)%	1-year 36%	1-year 94%	24 (0)%	0%
Hill, 2019 [53]	CD5	CD28	TNHL	≥2	5 *	44 (33)%	NR	NR	33 (0)%	11 (0)%
Zhang, 2023 [54]	CD7	4-1BB	TNHL MF	≥2	2 *	50 (0)%	NR	NR	80 (10)%	0 (0)%
Frigault, 2021 [55]	CD37	4-1BB	LBCL, CTCL, HL	≥2	4	75 (75)%	NR	NR	75 (25)%	25 (25)%
Cwynarski, 2023 [56]	TRBC1	4-1BB	TNHL	≥1	10	67 (56)%	NR	NR	40 (10)%	0 (0)%
Allogeneic CAR products										
ALLO-501 [57]	CD19	4-1BB	LBCL, FL	≥2	46	75 (50)%	NR	NR	22 (2)%	NR (0)%
ALLO-501/ALLO-501A [58]	CD19	4-1BB	LBCL	≥3	33	58 (42)%	NR	NR	24 (0)%	0 (0)%
PBCAR0191 [59]	CD19	NR	BNHL	≥2	13	77 (54)%	NR	NR	46 (0)%	31 (0)%
CTX110 [60]	CD19	NR	LBCL	≥2	32	56 (34)%	NR	NR	56 (0)%	9 (6)%
CTX130 [61]	CD70	NR	TNHL	≥1	15	71 (29)%	NR	NR	47 (0)%	20 (0)%
CB-010 [62]	CD19	NR	BNHL	≥2	6	5/6 (4/6) •	NR	NR	1 (1) •	1 (1) •
iC9/CAR.19/IL15-Transduced CB-NKs (cord blood-derived NK cells) [63]	CD19	CD28	LBCL ^†^	≥3	6 *	73 (64)%	NR	NR	0 (0)%	0 (0)%
RD13-01 [64]	CD7	4-1BB	TNHL ^†^	≥2	4	82 (64)%	NR	NR	83 (0)%	0 (0)%
CD30.CAR-EBVSTs (allo EBV-CTLs) [65]	CD30	CD28	HL	≥3	14	69 (38)%	NR	NR	29 (0)%	0 (0)% (NR)
NKX019 (allo CAR-NK) [66]	CD19	OX40	BNHLs ^†^	≥2	14	71 (57)%	NR	NR	26 (0)%	0 (0)%
FT516 (iPSC-derived NK cells) [67]	hnCD16	NR	BNHLs	≥2	6	3/4 (2/4) •	NR	NR	0 (0)%	0 (0)%
FT819-101 (iPSC-derived NK cells) [68]	CD19	NR	BNHLs	≥2	12 *	NR	NR	NR	8(0)%	0(0)%
AB-101 (cord blood-derived NK cells) [69]	CD16	NR	BNHLs	NR	17	67(50)%	NR	NR	12(0)%	0(0)%

° Sequential infusion of two different CAR-T products. * Preliminary data. • Absolute value among evaluable cases. † included in the basket trial. Abbreviations: ALCL: anaplastic large T-cell lymphoma, BNHLs: B-cell non-Hodgkin lymphoma, CLL: chronic lymphocytic leukemia, CR: complete response, CRS: cytokine release syndrome, CTCL: cutaneous T-cell lymphoma, DLBCL: diffuse large B-cell lymphoma, EATL: enteropathy-associated T-cell lymphoma, FL: follicular lymphoma, HL: Hodgkin lymphoma, I: intravenous route, ICANS: immune effector cell-associated neurotoxicity syndrome, I-VEN: intraventricular route, LBCL: large B-cell lymphoma m: months, MF: mycosis fungoides, MZL: marginal zone lymphoma, NK: natural killer, NR: not reported, OS: overall survival, ORR: overall response rate, PCNSL: primary central nervous system lymphoma, PFS: progression-free survival, R/R: relapsed/refractory, SS: Sezary syndrome, SCNSL: secondary nervous system lymphoma, TNHLs: T-cell non-Hodgkin lymphoma, y: year.

**Table 2:** Ongoing trials of novel products or in novel settings.

9	CAR-T Product	Target	Costimulatory Domain	Target Population	Study Phase	Line of Therapy (*n*)	Recruiting (Yes/No)
B-cell malignancies							
NCT05371093	Axi-cel	CD19	CD28	FL	III	>POD24/>2	yes
NCT05537766	Brexu-cel	CD19	CD28	WM, HCL	II	>1	no
NCT06043323	Axi-cel + RT	CD19	CD28	FL	II	>2	no
NCT05972720	CRG-022	CD22	NR	DLBCL	II	>1 *	no
NCT05091541	CT120	CD19/22	NR	DLBCL	I/II	>2	no
NCT05098613	CD19 × 22 CAR T	CD19/22	NR	BNHLs	I	>2	yes
NCT05797233		CD19/20	NR	BNHLs	I	variable	yes
NCT05607420	UCART20 × 22	CD20/22	NR	BNHLs	I	>2	yes
NCT03287817	AUTO3	CD/19/22	NR	DLBCL, PMBCL	I/II	>1 refractory/>2	no
NCT04989803	KITE-363 KITE-753	CD19/20	NR	BNHLs	I	>1	yes
NCT03870945	MB-CART2019.1	CD19/20	NR	BNHLs	I/II	variable	no
NCT04443829	AUTO1	CD19	4-1BB	PCNSL	I	>1	no
NCT05651178	NR	CD19/22	NR	BALL, BNHLs with CNSD	I	>1	yes
NCT04532203	NR	CD19	NR	BALL, BNHLs with CNSD	I	>1	yes
NCT05625594	ICV CAR-T	CD19	CD28	PCNSL	I	>1	yes
NCT04608487	axi-cel	CD19	CD28	PCNSL, SCNSL	I	>1	no
Hodgkin lymphoma, T-cell lymphomas							
NCT02917083	CD30.CAR-T	CD30	NR	HL, NHLs CD30+	I	>1	yes
NCT04008394	-	CD30	NR	HL, NHLs CD30+	I	>1	unknown
NCT04288726	Allo-CD30.CAR-EBVST	CD30	NR	HL, NHLs CD30+	I	>1	yes
NCT04268706	CD30.CAR-T	CD30	NR	HL	II	>2	no
NCT03383965	ICAR30-T	CD30	NR	HL, ALCL	I	>1	yes
NCT02690545	ATLCAR.CD30	CD30	NR	HL, NHLs CD30+	Ib/II	>3	yes
NCT05352828	CD30.CAR-T + Nivo	CD30	NR	HL	I	>1	no
NCT04653649	HSP-CAR30	CD30	4-1BB	HL, NHLs CD30+	I/II	>2	yes
NCT02259556	CART30	CD30	NR	HL, NHLs CD30+	I/II	>2	yes
NCT03602157	ATLCAR.CD30.CCR4	CD30, CCR4	NR	HL, CTCL, GZL	I	>2	yes
Allogenic CAR products							
NCT04176913	LUCRA-20S (Allo CAR-T)	CD20	NR	BNHLs	I	≥1 DLBCL ≥2 others BNHLs	no
NCT04384393	ThisCART19 (Allo CAR-T)	CD19	NR	BNHLs	I	NR (R/R)	yes
NCT04601181	ThisCART22 (Allo CAR-T)	CD22	NR	BNHLs	I	NR (R/R)	yes
NCT01430390	Allo EBV-CTLs	CD19	NR	BHLs	I	NR (R/R)	no
NCT02050347	CD19.CAR-CD28Z T (DDTC)	CD19	CD28	BNHL post AlloHSCT	I	NR (R/R post AlloHSCT)	no
NCT04026100	CTA101 (UCART)	CD19/CD22	NR	DLBCL	I	≥2	unknown
NCT03166878	UCART019	CD19	4-1BB	BNHLs	I/II	NR (R/R)	unknown
NCT04030195	PBCAR20A-01	CD20	NR	BNHLs	I/IIa	≥2	no
NCT04264078	CD7 UCAR-T cells	CD7	NR	T-cell malignancies	I	NR	unknown
NCT03774654	Allo CAR-NKT	CD19	NR	BNHLs	I	≥2	yes
NCT04245722	iDNKs	CD19	NR	BNHLs	I	NR (R/R)	no
NCT04629729	FT516 (iDNKs)	CD19	NR	BNHLs	I	≥2	yes
NCT02892695	anti-CD19 CAR-NK	CD19	NR	BNHLs	I/II	NR (R/R)	unknown
NCT04887012	HLA haplo CAR-NK	CD19	NR	BNHLs	I	≥2	yes
NCT04639739	anti-CD19 CAR NK	CD19	NR	BNHLs	I	≥1	no
NCT02656147	CD19 CAR γδT-cells	CD19	NR	BNHLs	I	NR (R/R)	unknown

* After anti-CD19 CAR-T failure. Abbreviations: ALCL: anaplastic large T-cell lymphoma, BALL: B-cell acute lymphoblastic leukemia, BNHLs: B-cell non-Hodgkin lymphomas, CNSD: central nervous system disease, CTCL: cutaneous T-cell lymphoma, DDTC: donor-derived T-cells, EBVST: Epstein–Barr virus-specific T cells, FL: follicular lymphoma, GZL: grey-zone lymphoma, haplo: haploidentical, HCL: hairy cell leukemia, HL: Hodgkin lymphoma, ICV: intracerebroventricularly administered, iDNKs: iPSC-derived NK cells, LBCL: large B-cell lymphoma, MCL: mantle cell lymphoma, NCT: national clinical trial; Nivo: nivolumab, NR: not reported, PCNSL: primary central nervous system lymphoma, SCNSL: secondary nervous system lymphoma, WM: Waldenstrom macroglobulinemia.
